# Machine learning reveal shared diagnostic biomarkers and convergent pathways in age-related hearing loss and sarcopenia

**DOI:** 10.1097/MD.0000000000045306

**Published:** 2025-10-24

**Authors:** Ming Li

**Affiliations:** a Department of Otolaryngology, Shangyu People’s Hospital of Shaoxing, Shaoxing University, Shaoxing, Zhejinag Province, China.

**Keywords:** age-related hearing loss, diagnostic biomarkers, hub genes, machine learning, sarcopenia

## Abstract

Age-related hearing loss (HL) and sarcopenia (ARS) are prevalent geriatric syndromes sharing common risk factors. This study aimed to identify shared biomarkers and elucidate convergent pathogenic mechanisms. Transcriptomic datasets were obtained from public database. Differential expression analysis was performed, followed by enrichment analysis. Hub genes were identified via LASSO regression, SVM-RFE, and random forest algorithms. Diagnostic performance was evaluated using receiver operating characteristic curve analysis across 6 independent cohorts. Comprehensive integrative analysis revealed distinct yet overlapping molecular signatures between HL and ARS. In HL, 11 upregulated and 16 downregulated genes were shared between 2 diseases, and complement and coagulation cascades, Toll-like receptor signaling, efferocytosis, as well as immune response processes were found to be associated with these genes. Machine learning identified 10 hub genes (*AIMP2, JUN, SEMA5A, RASL12, GUSB, C1QA, GYPC, IRF7, C1QB, SERPING1*) as shared biomarkers. Notably, these genes demonstrated robust diagnostic utility: individual genes exhibited area under the curve (AUC) values > 0.7 in most cohorts. Although the combined 10-gene model achieved AUC = 1 in several cohorts, these results should be interpreted with caution due to the limited sample sizes in some datasets (e.g., GSE6045, n = 3 per group), which may inflate performance metrics. Permutation tests confirmed that the AUC values were significantly better than chance in several cohorts (*P* < .05). This study pioneers a machine-learning framework to uncover shared molecular drivers of HL and ARS, identifying 10 hub genes as promising diagnostic biomarkers.

## 1. Introduction

Age-related hearing loss (ARHL) and sarcopenia represent 2 critical geriatric syndromes with significant global health burdens. ARHL, characterized by progressive hearing impairment due to cochlear degeneration and neural pathway alterations, affects 60% of individuals aged 65 to 75 years, severely impairing communication and quality of life.^[1]^ Concurrently, sarcopenia – the age-related loss of skeletal muscle mass, strength, and function – is prevalent in 5% and 10% in the general population and independently associated with falls, fractures, and mortality.^[2]^ Emerging evidence suggests an epidemiological overlap between these conditions; for instance, a prospective cohort study of 4603 Chinese middle-aged and elderly adults revealed a 35.4% increased risk of hearing loss (HL) among individuals with sarcopenia.^[3]^ Despite this correlation, the shared biological mechanisms and potential biomarkers underlying their co-occurrence remain poorly understood, hindering the development of integrated diagnostic and therapeutic strategies.

While ARHL and sarcopenia share common risk factors such as oxidative stress, mitochondrial dysfunction, and chronic inflammation,^[4,5]^ their pathophysiological interplay is complex. For example, auditory pathway degeneration in ARHL may disrupt neural-muscular crosstalk, exacerbating muscle wasting.^[6]^ Conversely, sarcopenia-induced physical inactivity could accelerate auditory system decline through reduced neurotrophic factor secretion.^[7,8]^ Current studies predominantly employ single-omics approaches (e.g., transcriptomics or proteomics) to identify disease-specific markers, but these methods often overlook cross-disease synergies. Furthermore, traditional statistical models like logistic regression struggle to capture nonlinear interactions among high-dimensional variables, limiting their utility in uncovering shared molecular signatures.

Machine learning (ML) algorithms, particularly ensemble methods like XGBoost and random forests, have revolutionized biomedical research by enabling high-dimensional data integration and nonlinear relationship modeling. For instance, XGBoost demonstrated an area under the curve (AUC) of 0.872 in predicting malnutrition in elderly cancer patients by analyzing 7 variables, including diabetes status and lymphocyte count.^[9]^ Similarly, RNA sequencing combined with ML identified differential expression patterns (e.g., *C1QA* and *TPPP3*) predictive of sarcopenia with an AUC of 0.7.^[10]^ These advancements underscore ML’s potential to identify “bridge” biomarkers that transcend single-disease boundaries.

**Figure 1.**
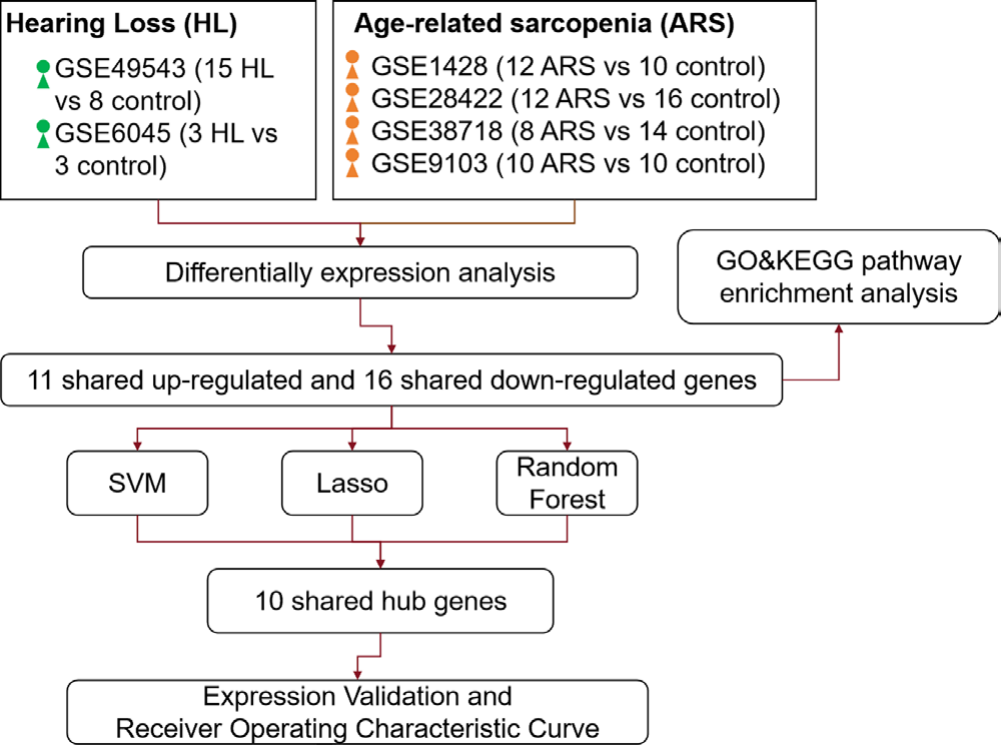
Flowchart for identifying shared biomarkers and mechanisms between HL and ARS. ARS = age-related sarcopenia, HL = hearing loss.

Presently, most research focuses on isolated disease mechanisms, neglecting cross-condition interactions. Candidate biomarkers often lack independent validation in diverse cohorts. Small sample sizes and heterogeneous data sources hinder robust model generalization. Therefore, our study employs a multi-algorithm framework to systematically identify shared biomarkers between ARHL and sarcopenia. We hypothesize that integrating 2 disease-related genes using ML will reveal conserved pathways, such as mitochondrial dysfunction or inflammatory signaling, that drive both conditions. Figure 1 illustrates the flowchart of this study.

## 2. Materials and methods

### 2.1. Data acquisition and preprocessing

Raw transcriptomic data for HL (GSE6045, GSE49543) and age-related sarcopenia (ARS; GSE1428, GSE9103, GSE28422, GSE38718) were downloaded from the Gene Expression Omnibus (GEO, https://www.ncbi.nlm.nih.gov/geo/) database. To integrate data from multiple platforms and species (human and mouse), the following standardization and harmonization steps were applied: Probe annotation and gene mapping: platform-specific annotation files were used to map probes to official gene symbols. For genes matched by multiple probes, the median expression value was taken as the representative expression level. Low-abundance gene filtering: genes with expression levels below the 75th percentile across all samples were excluded to minimize noise. Batch correction and normalization: the normalizeBetweenArrays function in the limma R package was used for quantile normalization to ensure comparable distributions across samples. Normalized data were log₂-transformed to stabilize variance. Batch effects were corrected using the ComBat algorithm from the sva package. The effectiveness of batch correction was visually assessed via principal component analysis before and after adjustment. Cross-species gene homolog mapping: the homologene R package was used to map orthologous genes between human and mouse based on conserved gene symbols and functional annotations. Only genes with one-to-one orthologous relationships and consistent functional annotations were retained to avoid misinterpretation. There is no need for informed consent in our study since the unidentified data were free from medical ethics review.

### 2.2. Differential expression analysis

Limma was employed to identify differentially expressed genes (DEGs) between HL and ARS cohorts. A linear model was fitted using the lmFit function, followed by empirical Bayes moderation of variances via eBayes. DEGs were defined as genes with log2-fold change (logFC) > 0.2 and *P*-values < .05. Upregulated and downregulated genes were separately annotated, and volcano plots were generated using ggplot2, with logFC on the x-axis and −log10(*P*-value) on the y-axis. Heatmaps of representative DEGs were visualized using pheatmap, with rows normalized per gene and columns grouped by disease status. No sample clustering was applied to avoid bias.

### 2.3. Functional enrichment analysis

Orthologous gene mapping between murine and human datasets was performed using the homologene R package to ensure cross-species comparability. Common upregulated and downregulated genes were extracted from DEG lists of both diseases. Gene ontology (GO) enrichment (biological process, cellular component, molecular function) and Kyoto encyclopedia of genes and genomes (KEGG) pathway analysis were conducted via clusterProfiler. Top 10 enriched terms were depicted in bubble charts, with significance (−log10(*P*-value)) versus fold enrichment. Venn diagrams were generated to illustrate intersections of GO terms and pathways between HL and ARS.

### 2.4. ML-based core gene identification

Three computational methods were implemented for feature selection: LASSO regression (glmnet package) with 10-fold cross-validation to determine the optimal λ value, producing sparse gene coefficients; SVM-RFE (mRFE package) using a linear kernel and recursive feature elimination, with mean decrease in accuracy as the ranking criterion; Random Forest (randomForest package) with 500 trees, evaluating variable importance via mean decrease in Gini index. Venn diagrams integrated overlapping genes from all 3 methods, with their union defined as core candidate genes. Intersection of core genes between HL and ARS was performed to prioritize shared therapeutic targets.

### 2.5. Expression validation and ROC analysis

Differential expression of core genes was validated using boxplots with Wilcoxon rank-sum tests (*P* < .05). Receiver operating characteristic (ROC) curves were generated for each gene using the pROC package. A combined logistic regression model incorporating all core genes was fitted to evaluate joint predictive performance. To ensure robust performance evaluation and mitigate overfitting concerns, especially in small sample size cohorts, we implemented a comprehensive validation framework: 1) Cross-Validation: Model stability was assessed through stratified k-fold cross-validation (k = 5) for datasets with fewer than 20 samples. Cross-validated AUC values were reported alongside training AUC. 2) Permutation Testing: Statistical significance of model performance was evaluated through 1000-label permutation tests, generating empirical null distributions and calculating *P*-values for the observed AUC values. The GSE6045 cohort was not subjected to permutation tests due to having fewer than 10 samples.

## 3. Results

### 3.1. Identification of DEGs in HL

**Figure 2.**
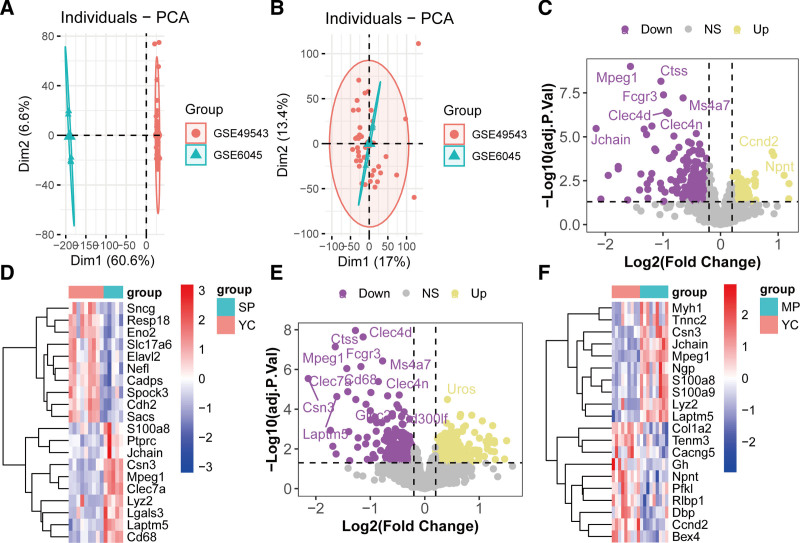
Identification of DEGs in HL. (A) PCA of the GSE49543 and GSE60445 cohorts before batch effect removal. (B) PCA after batch effect adjustment. (C) Volcano plot of DEGs between the SP and YC groups in the GSE49543 cohort. (D) Heatmap of aberrantly expressed genes in the SP versus YC comparison within the GSE49543 cohort. (E) Volcano plot of DEGs between the MP and YC groups in the GSE49543 cohort. (F) Heatmap of aberrantly expressed genes in the MP versus YC comparison within the GSE49543 cohort. DEG = differentially expressed genes, HL = hearing loss, MP = mild presbycusis, PCA = principal component analysis, SP = severe presbycusis, YC = young controls.

Batch effects in the HL cohort were mitigated using ComBat, as evidenced by improved clustering of samples postcorrection (Fig. 2A and B). Differential expression analysis was performed on the GSE49543 dataset, which included 4 groups: young controls (YC) with good hearing (YC , n = 8), middle-aged controls (MA, n = 17), mild presbycusis (MP, n = 9), and severe presbycusis (SP, n = 6). Comparing MP versus YC groups identified 153 upregulated and 210 downregulated genes (Fig. 2C and D, Table S1, Supplemental Digital Content, https://links.lww.com/MD/Q366). Similarly, SP versus YC comparisons revealed 287 upregulated and 254 downregulated genes (Fig. 2E and F, Table S2, Supplemental Digital Content, https://links.lww.com/MD/Q366). Hierarchical clustering of heatmaps highlighted distinct expression patterns for key genes across groups.

### 3.2. DEG identification in ARS

**Figure 3.**
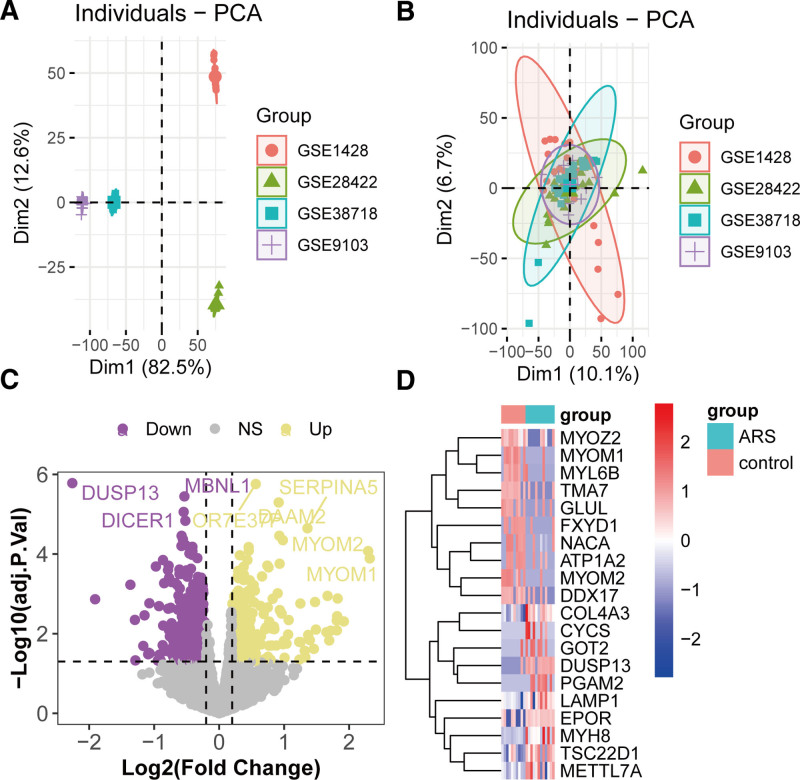
Identification of aberrantly expressed genes in ARS. (A) PCA of the GSE1428 cohort before batch effect removal. (B) PCA after batch effect adjustment. (C) Volcano plot of DEGs between the ARS and control groups in the GSE1428 cohort. (D) Heatmap of the top 20 differentially expressed genes in ARS. ARS = age-related sarcopenia, DEGs = differentially expressed genes, PCA = principal component analysis.

Batch effects across the 4 ARS cohorts were mitigated using ComBat, as demonstrated by improved sample clustering and reduced technical variability in principal component analysis plots (Fig. 3A and B). For the GSE1428 cohort, which included global gene expression profiles of the vastus lateralis muscle from 10 young males (19–25 years) and 12 older males (70–80 years), differential expression analysis identified 369 upregulated and 531 downregulated genes in ARS (older) compared to YC (Fig. 3C, Table S3, Supplemental Digital Content, https://links.lww.com/MD/Q366). Genes were filtered using a threshold of |log2-fold change (logFC)| > 0.2 and *P*-values < .05. The top 10 most significantly altered genes, including muscle-specific proteins *MYOZ2, MYOM1*, and *MYL6B*, are highlighted in Figure 3D.

### 3.3. Shared upregulated genes between HL and ARS

**Figure 4.**
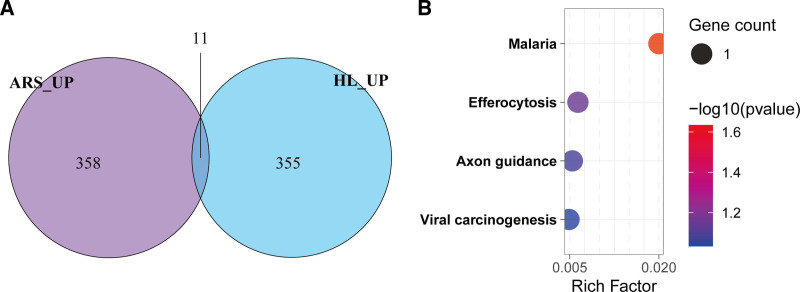
Consistently upregulated genes and associated pathways in HL and ARS. (A) Venn diagram showing overlapping upregulated genes between ARS and HL. (B) KEGG pathway enrichment analysis of 11 shared upregulated genes. ARS = age-related sarcopenia, HL = hearing loss, KEGG = Kyoto Encyclopedia of Genes and Genomes.

Following differential expression analysis, the union of upregulated genes identified from MP and SP datasets yielded 369 genes in HL. A Venn diagram analysis further revealed 11 overlapping upregulated genes between HL and ARS (Fig. 4A), including *AIMP2, SLC16A1, DNAJA3, GYPC, DEXI, MSL3, RASL12, UQCC1, MTIF2, SLCO5A1*, and *SEMA5A*. GO enrichment analysis did not identify statistically significant terms (adjusted *P* > .05; data not shown). However, KEGG pathway enrichment demonstrated that these shared genes were significantly enriched in malaria, efferocytosis, axon guidance, and viral carcinogenesis pathways (Fig. 4B).

### 3.4. Shared downregulated genes between HL and ARS

**Figure 5.**
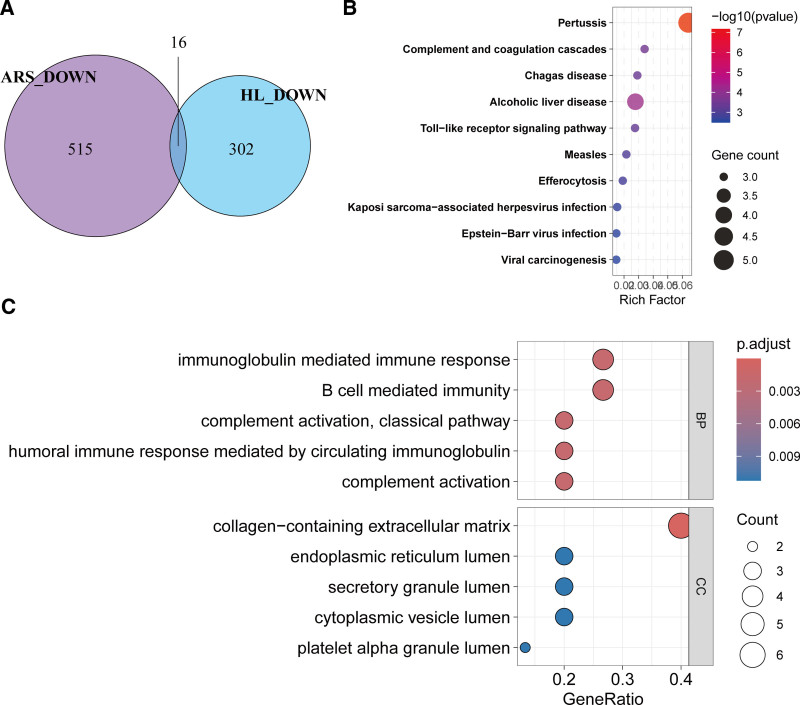
Consistently downregulated genes, pathways, and biological processes in HL and ARS. (A) Venn diagram of overlapping downregulated genes between ARS and HL. (B) KEGG pathway enrichment analysis and (C) GO enrichment analysis of biological processes and cellular components for 16 shared downregulated genes. ARS = age-related sarcopenia, GO = gene ontology, HL = hearing loss, KEGG = Kyoto Encyclopedia of Genes and Genomes.

Further analysis of the union of MP and SP datasets identified 531 downregulated genes in HL. A comparative Venn analysis revealed 16 genes significantly downregulated in both HL and ARS (Fig. 5A), including *JUN, CD14, C1QA, TIMP1, C1QB, SERPING1, LTBP2, KIAA0907, SFMBT1, MFGE8, IRF7, SGPL1, PLP2, ARID1A, GUSB*, and *CCND1*. KEGG pathway enrichment analysis demonstrated that these genes were significantly enriched in immunological pathways, including the complement and coagulation cascades, Toll-like receptor signaling, and efferocytosis (Fig. 5B). GO biological process enrichment further highlighted their involvement in immune regulation, such as immunoglobulin-mediated immune response, B cell-mediated immunity, and classical complement activation (Fig. 5C). These findings collectively suggest dysregulated immune and coagulation mechanisms in HL and ARS pathogenesis.

### 3.5. ML identification of hub genes

**Figure 6.**
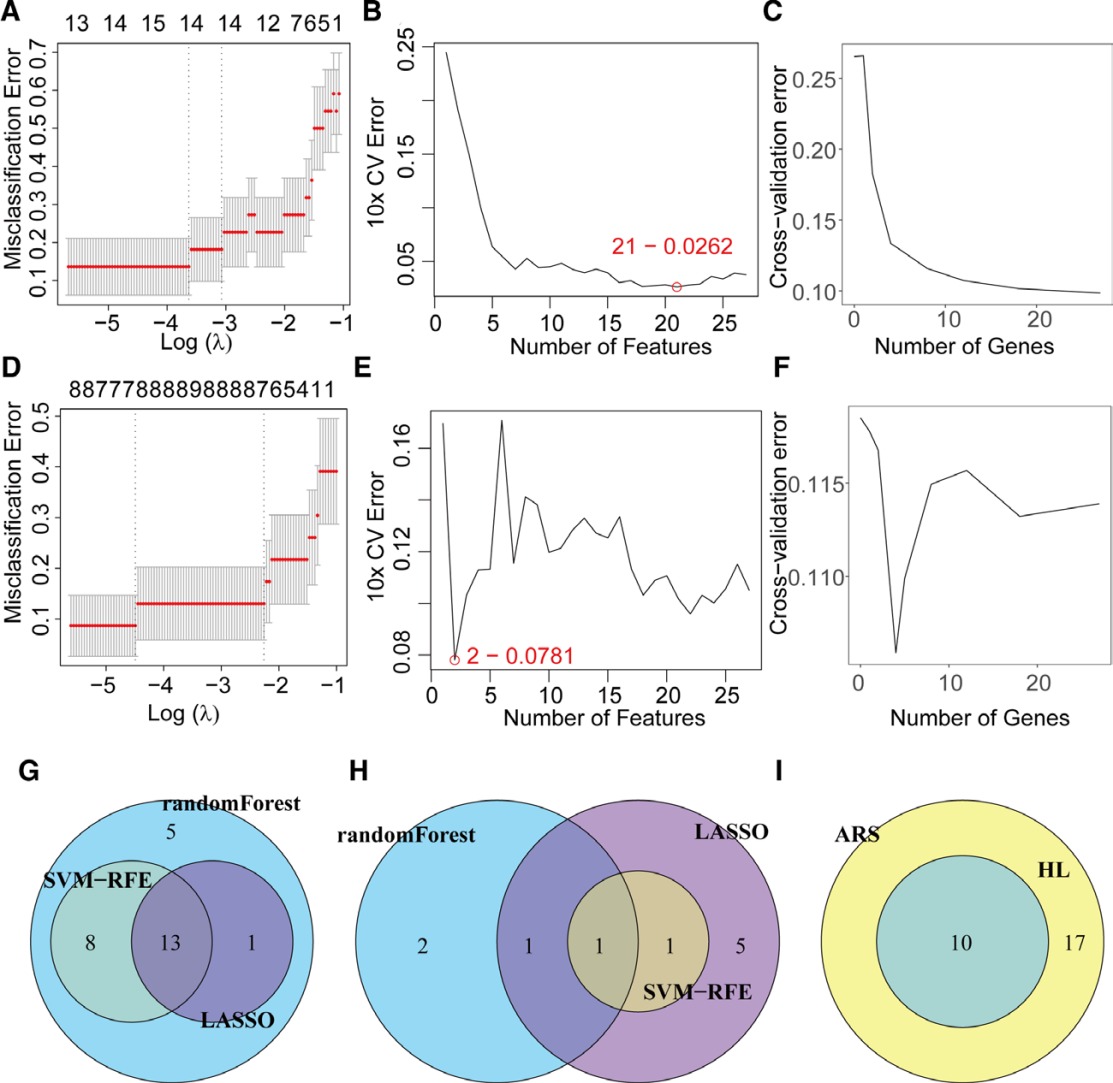
Identification of hub genes via machine learning algorithms. (A–C) LASSO, SVM, and Random Forest algorithms identified 14, 21, and 26 core genes in ARS, respectively. (D–F) LASSO, SVM, and Random Forest identified 8, 2, and 4 hub genes in HL, respectively. (G) Venn diagram of overlapping hub genes in ARS across algorithms. (H) Venn diagram of overlapping hub genes in HL across algorithms. (I) Venn diagram of shared hub genes between ARS and HL. ARS = age-related sarcopenia, HL = hearing loss.

In this study, we employed 3 ML algorithms – LASSO regression, SVM, and random forest – to identify hub genes and shared hub genes between HL and ARS. In ARS, LASSO, SVM, and random forest identified 14, 21, and 26 core genes, respectively (Fig. 6A–C, Table S4, Supplemental Digital Content, https://links.lww.com/MD/Q366). For HL, the algorithms detected 8, 2, and 4 hub genes, respectively (Fig. 6D–F, Table S5, Supplemental Digital Content, https://links.lww.com/MD/Q366). Venn diagram analysis revealed 27 unique hub genes in ARS (Fig. 6G) and 10 in HL (Fig. 6H). Notably, the intersection of these hub gene sets identified 10 overlapping genes shared between HL and ARS (Fig. 6I), including *AIMP2, JUN, SEMA5A, RASL12, GUSB, C1QA, GYPC, IRF7, C1QB*, and *SERPING1*.

### 3.6. Expression validation of hub genes

**Figure 7.**
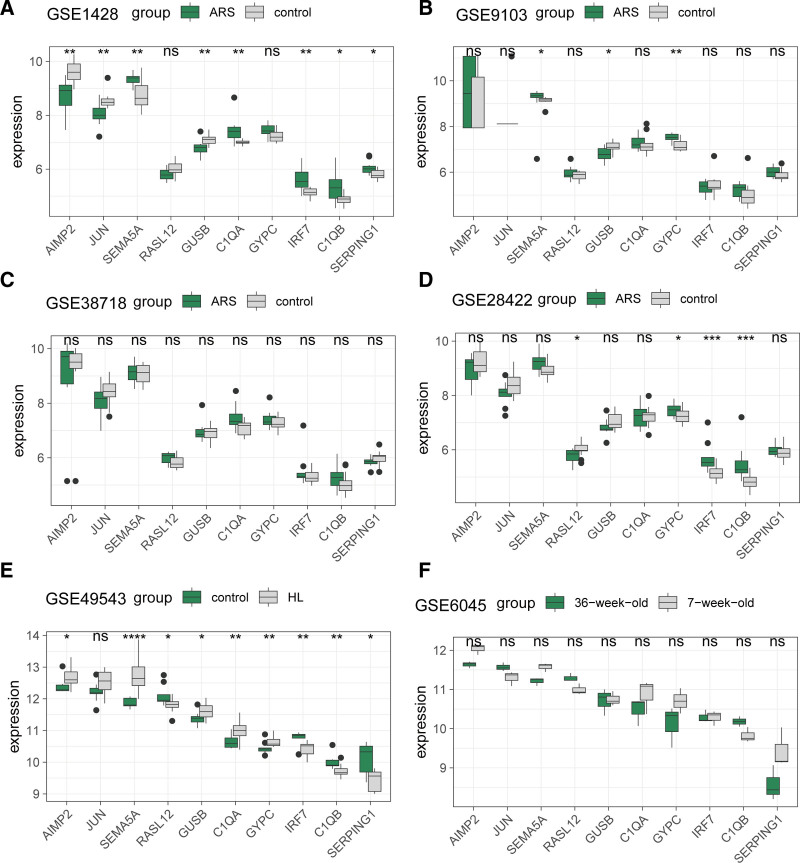
Expression analysis of 10 hub genes across multiple cohorts. Expression levels of the 10 hub genes in (A) GSE1428, (B) GSE9103, (C) GSE38718, (D) GSE28422, (E) GSE49543, and (F) GSE6045.

We conducted a systematic expression analysis of the 10 hub genes across all cohorts. In ARS patients from GSE1428, *AIMP2, JUN*, and *GUSB* showed significant downregulation compared to controls (*P* < .05), while *SEMA5A, C1QA, IRF7, C1QB*, and *SERPING1* were upregulated (Fig. 7A). In GSE9103 (ARS), only *GUSB* was downregulated, whereas *SEMA5A* and *GYPC* were upregulated (Fig. 7B). No significant differential expression was observed in GSE38718 (ARS; Fig. 7C). In GSE28422 (ARS), *RASL12* was downregulated, while *GYPC, IRF7*, and *C1QB* were upregulated (Fig. 7D). For HL cohorts, GSE49543 revealed 5 upregulated genes (*AIMP2, SEMA5A, GUSB, C1QA, GYPC*) and 4 downregulated genes (*RASL12, IRF7, C1QB, SERPING1*) compared to controls (*P* < .05; Fig. 7E). However, no significant differences were detected in GSE6045 (HL), likely attributable to insufficient sample size (n = 3; Fig. 7F).

### 3.7. Diagnostic performance of hub genes

**Figure 8.**
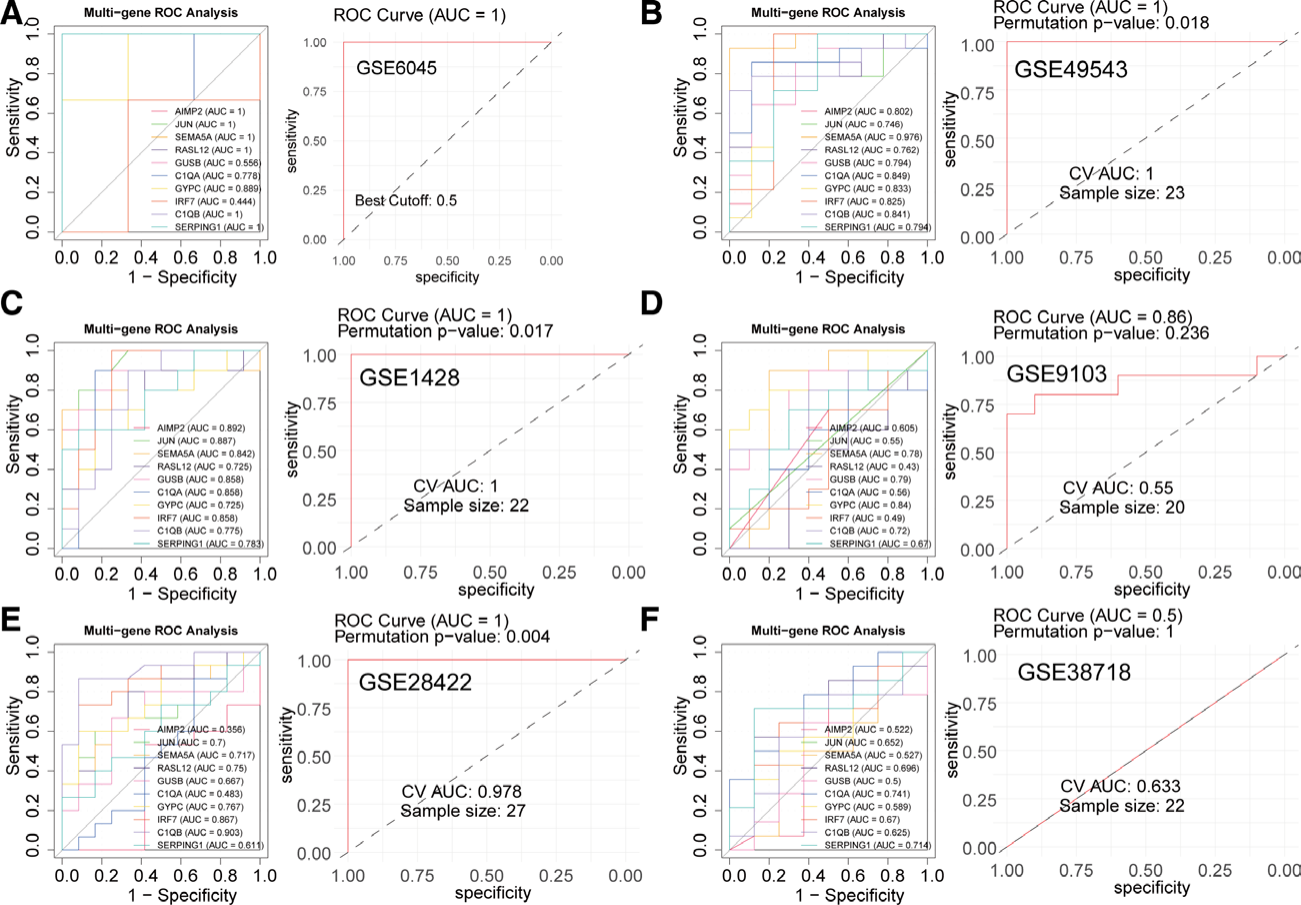
Diagnostic performance evaluation of 10 hub genes and their combinations in HL and ARS. Diagnostic efficacy of individual and combined models in (A) GSE6045, (B) GSE49543, (C) GSE1428, (D) GSE9103, (E) GSE28422, and (F) GSE38718 cohorts. ARS = age-related sarcopenia, HL = hearing loss.

To evaluate the diagnostic performance of the 10 hub genes in HL and ARS, ROC curve analysis was performed across all cohorts. In GSE6045 (HL), all genes except *GUSB* and *IRF7* exhibited excellent diagnostic accuracy (AUC > 0.7), with the 10-gene combined model achieving perfect discrimination (AUC = 1; Fig. 8A). Similarly, in GSE49543 (HL), all individual genes and the combined model showed strong diagnostic performance (AUC > 0.7; AUC = 1 for the model; Fig. 8B). For ARS cohorts, GSE1428 demonstrated robust AUC values (>0.7) for all genes, with the combined model reaching CV AUC = 1 (Fig. 8C). In GSE9103 (ARS), *SEMA5A* (AUC = 0.78), *GUSB* (AUC = 0.79), *GYPC* (AUC = 0.84), *C1QB* (AUC = 0.72), and the combined model (CV AUC = 0.55) all achieved clinically relevant diagnostic accuracy (Fig. 8D). In GSE28422 (ARS), *JUN, SEMA5A, RASL12, GYPC, C1QB*, and the combined model showed strong performance (AUC > 0.7; Fig. 8E). While GSE38718 (ARS) identified *C1QA* (AUC = 0.81) and *SERPING1* (AUC = 0.79) as reliable biomarkers, the combined model achieved only limited accuracy (CV AUC = 0.633; Fig. 8F). To evaluate the statistical significance of the ROC results, permutation tests were performed by randomly shuffling the class labels 1000 times. The empirical *P*-values for the AUC of the combined model were below 0.05 in the GSE1428, GSE28422, GSE49543 cohorts, confirming that the performance was significantly better than random chance (Fig. 8B–F).

## 4. Discussion

The present study elucidates the molecular interplay between HL and ARS, 2 prevalent geriatric syndromes, through a multi-omics and ML-driven approach. Our findings reveal shared pathological pathways and diagnostic biomarkers, providing novel insights into the convergence of aging-related disorders. While our study identifies a robust transcriptomic signature, its translation into clinical practice requires a clear and deliberate pathway. The immediate next step involves technical and analytical validation in larger, independent prospective cohorts. A well-designed prospective observational study should recruit a diverse population reflecting real-world heterogeneity in age, sex, comorbidities (e.g., diabetes, cardiovascular disease), and lifestyle factors. This is critical to assess the generalizability of the 10-gene panel and to determine if its performance is confounded by common age-related conditions. Such a study would measure biomarker expression (via qPCR or eventually a targeted RNA-seq panel) alongside gold-standard measures of hearing (audiometry) and sarcopenia (DEXA scans, grip strength). This allows for the development of a multivariate clinical prediction model that integrates both molecular and clinical variables.

Previous studies have independently explored HL and ARS, yet few have addressed their molecular overlap. Epidemiological evidence suggests a bidirectional association between auditory and musculoskeletal decline, with sarcopenia increasing the risk of hearing impairment by 35.4% in middle-aged and elderly adults.^[3]^ Mechanistically, oxidative stress and mitochondrial dysfunction are implicated in both diseases. Mitochondrial DNA mutations in cochlear cells drive HL progression,^[5]^ while mitochondrial fragmentation in muscle fibers accelerates sarcopenia.^[11]^ Our KEGG pathway analysis identified “malaria” and “efferocytosis” as shared pathways, which may reflect dysregulated innate immunity and cellular clearance mechanisms – a hypothesis supported by studies linking chronic inflammation to both HL^[12]^ and sarcopenia.^[13]^

The shared molecular signatures between HL and ARS revealed by KEGG and GO enrichment analyses provide critical insights into their convergent pathophysiology. Enrichment in pathways such as efferocytosis, complement and coagulation cascades and Toll-like receptor signaling highlights systemic immune dysregulation as a core mechanism. Efferocytosis, the process by which phagocytes clear apoptotic cells, plays a pivotal role in maintaining tissue homeostasis and preventing chronic inflammation, with its dysregulation implicated in both sensorineural HL and sarcopenia.^[14]^ In the inner ear, defective efferocytosis of apoptotic hair cells due to impaired recognition signals (e.g., phosphatidylserine externalization) or phagocyte dysfunction (e.g., MerTK deficiency) leads to the accumulation of cellular debris,^[15]^ triggering cGAS-STING and NLRP3 inflammasome activation,^[16]^ which exacerbates cochlear inflammation and hair cell apoptosis.^[17,18]^ Similarly, in skeletal muscle, failed clearance of apoptotic myocytes perpetuates M1 macrophage polarization via Gas6/Axl signaling, promoting pro-inflammatory cytokine release (e.g., IL-6, TGF-β) that suppresses satellite cell activity while driving fibrosis and proteolysis.^[19,20]^ Cross-tissue mechanisms involve oxidative stress amplification through NADPH oxidase activation^[21]^ and mitochondrial DNA leakage,^[22]^ which activate NF-κB and JAK/STAT pathways, creating a feedforward loop of inflammation and tissue damage.^[23]^ Therapeutic strategies targeting efferocytosis, such as TAM receptor agonists (e.g., Gas6 supplementation) or metabolic reprogramming via PPARγ/AMPK activation, show promise in preclinical models by enhancing apoptotic cell clearance, reducing pro-inflammatory mediators, and restoring mitochondrial function.^[24,25]^ These insights highlight efferocytosis as a unifying therapeutic target for mitigating both auditory and muscular degeneration through inflammation resolution and metabolic restoration.

Notably, complement and coagulation cascades were prominently enriched in low-expressed shared genes. The complement cascade, activated via classical, lectin, or alternative pathways, releases pro-inflammatory mediators such as C3a and C5a, which recruit macrophages and neutrophils to the cochlea and skeletal muscle, perpetuating neuroinflammation and proteolysis.^[26]^ In the cochlea, macrophage activation disrupts the blood-labyrinth barrier, exacerbating oxidative stress through NADPH oxidase-dependent reactive oxygen species (ROS) production and mitochondrial DNA damage,^[27]^ while C3a/C5a binding to their receptors activates NLRP3 inflammasomes, driving interleukin-1β (IL-1β) and tumor necrosis factor-α (TNF-α) release that accelerates hair cell apoptosis and spiral ganglion neuron degeneration.^[28,29]^ Clinically, reduced serum C3 and C4 levels in idiopathic sudden SNHL inversely correlate with hearing thresholds, suggesting compensatory consumption due to hyperactivation or impaired regulation by inhibitors like factor H.^[30]^ Concurrently, coagulation dysregulation, characterized by elevated fibrinogen and D-dimer, promotes microthrombosis in cochlear and muscle microvasculature, reducing perfusion and oxygen delivery^[31,32]^; increased plasma viscosity correlates with HL severity and muscle atrophy,^[33,34]^ while impaired fibrinolysis due to antithrombin III deficiency delays tissue repair by permitting fibrin deposition that obstructs regenerative processes.^[35]^ Crosstalk between these systems amplifies pathology: complement-derived ROS and coagulation-related oxidative stress induce mtDNA mutations and NAD + depletion, activating JAK/STAT and MAPK pathways to exacerbate apoptosis in hair cells and myocytes.^[36]^ Furthermore, C3a/C5a-stimulated TGF-β/Smad signaling drives fibrosis in muscle,^[36]^ while anticoagulant deficiencies exacerbate microvascular endothelial dysfunction, impairing satellite cell activation and muscle regeneration. Mitochondrial ROS, a shared downstream effector, depletes ATP and activates AMPK/PKCζ,^[37]^ further suppressing PGC-1α-mediated mitochondrial biogenesis^[38]^ and insulin signaling via IRS-1 serine phosphorylation,^[39]^ creating a feedforward loop of metabolic collapse. Therapeutic strategies targeting this axis – such as complement inhibitors (anti-C5a), thrombolytics (rt-PA), Nrf2-activated ROS scavengers, and PGC-1α agonists – show promise in preclinical models by mitigating inflammation, restoring microcirculation, and enhancing mitochondrial function. This paradigm positions the complement-coagulation-mitochondria network as a critical therapeutic frontier for age-related degenerative disorders, warranting translational investigations into combinatorial interventions to disrupt their synergistic pathogenicity.

Toll-like receptors are central pattern recognition receptors of the innate immune system, activating downstream inflammatory and immune responses by recognizing pathogen-associated molecular patterns or damage-associated molecular patterns.^[40]^ Their canonical signaling pathways include the MyD88-dependent pathway (activating NF-κB and MAPK pathways) and the TRIF-dependent pathway (inducing type I interferons).^[41]^ Aberrant activation of Toll-Like Receptors is closely associated with chronic inflammation, autoimmune diseases, and tissue damage. Studies have shown that TLR4 is highly expressed in the cochlear Corti organ, and noise trauma or ototoxic drugs like cisplatin can activate TLR4, triggering the release of pro-inflammatory cytokines such as IL-6 and TNF-α, leading to hair cell apoptosis and spiral ganglion damage.^[42]^ Moreover, TLR4 activation upregulates NADPH oxidase via the NF-κB pathway, increasing ROS production and exacerbating cochlear oxidative damage.^[43]^ After TLR3 recognizes viral double-stranded RNA, it activates the TRIF-dependent pathway, inducing interferon secretion, but overactivation may lead to hair cell death via the caspase-3 pathway.^[44]^ In sarcopenia, gut microbiota dysbiosis leads to lipopolysaccharide entering the bloodstream, activating TLR4 in skeletal muscle.^[45]^ This activation promotes the expression of the ubiquitin-proteasome system (such as MuRF1 and MAFbx) through TLR4/MyD88/NF-κB and TLR4/AKT/FOXO pathways, accelerating muscle protein breakdown.^[46]^ TLR4 activation also inhibits the PI3K/Akt pathway,^[47]^ reducing the key muscle synthesis factor IGF-1 and worsening sarcopenia.^[48]^ These findings reveal TLR-mediated immunometabolic crosstalk as a unifying mechanism for comorbid neurosensory and musculoskeletal degeneration, providing a framework for combinatorial therapies targeting TLR signaling.

Our ML pipeline identified 10 hub genes (*AIMP2, JUN, SEMA5A*, etc) as potential mediators of these shared pathways. Notably, *JUN* and *SERPING1* emerged as key regulators of inflammatory responses. *JUN*, a component of the AP-1 transcription factor complex, is downregulated in muscle atrophy.^[49,50]^ Additionally, c-Jun phosphorylation serves as a biomarker of cochlear stress and mediates outer hair cell death via a paracrine mechanism. Inhibition of JNK or suppression of upstream activators of the JNK pathway confers cytoprotection to hair cells.^[51,52]^ Further supporting its role in shared pathophysiology, recent studies highlight JUN as a critical node in stress-induced apoptosis and inflammation across both auditory and musculoskeletal systems. For instance, JUN is implicated in the JNK/c-Jun signaling cascade, which is activated in response to oxidative stress, ototoxic drugs, and acoustic trauma in the cochlea.^[53,54]^ In sarcopenia, JUN expression is modulated in response to muscle disuse and denervation, and its downregulation is associated with muscle wasting.^[49,50]^ Notably, JUN is also identified as a key transcriptional regulator in multi-omics studies of sarcopenia, influencing pathways such as oxidative phosphorylation and inflammation.^[55]^ This dual involvement underscores JUN’s role as a convergence point for stress response pathways that drive cellular apoptosis and metabolic dysregulation in both tissues.

SERPING1, which encodes the C1 esterase inhibitor, is a critical regulator of the complement system and the contact activation pathway (kallikrein-kinin system). Our findings of its downregulation in both HL and ARS align with its role as a broad-spectrum inhibitor of inflammation. In the context of aging and tissue degeneration, diminished SERPING1 activity can lead to uncontrolled activation of complement cascades and excessive bradykinin production.^[56]^ This is particularly relevant as bradykinin increases vascular permeability and promotes pro-inflammatory signaling, mechanisms implicated in both cochlear injury and muscle wasting.^[57]^ Furthermore, recent evidence links SERPING1 dysregulation directly to musculoskeletal pathology. In Duchenne muscular dystrophy and other muscle atrophy models, SERPING1 was identified as a top hub gene, with its expression upregulated in dystrophic muscle, possibly as a compensatory response to intense inflammation and complement activation.^[58,59]^ Conversely, in ischemic muscle models, epigenetic dysregulation such as promoter hypomethylation can lead to altered SERPING1 expression, skewing macrophages towards a pro-inflammatory (M1) phenotype and impairing tissue repair.^[60]^ This suggests that SERPING1 malfunction, whether through decreased expression or failed compensatory upregulation, disrupts immune homeostasis, creating a permissive environment for chronic inflammation that exacerbates both auditory hair cell apoptosis and myocyte degradation.

IRF7, a master regulator of type I interferon responses, emerges as a pivotal shared gene in both ARHL and sarcopenia, implicating its role in immunometabolic crosstalk across these conditions. In zebrafish, IRF7 is essential for hair cell development, and its knockdown leads to hair cell loss and neuromast defects, suggesting a conserved role in auditory system integrity and positioning it within the HL gene family.^[61]^ Beyond developmental roles, IRF7 is induced in cochlear cells following acoustic trauma, where it contributes to innate immune activation and inflammatory signaling, potentially exacerbating sensory cell damage.^[62]^ In sarcopenia, IRF7 has been identified as a hub gene in aged muscle transcriptomes, particularly within a subset of interferon-responsive macrophages that are critical for satellite cell proliferation and muscle regeneration.^[63]^ Age-related decline in IRF7 expression within these macrophages impairs CXCL10-mediated activation of satellite cells, thereby compromising muscle repair and promoting fibrosis.^[63]^ Moreover, IRF7 overexpression has been linked to mitochondrial dysfunction and metabolic dysregulation in aging stromal cells, further connecting it to the energetic decline observed in both sarcopenia and ARHL.^[62]^ These findings collectively position IRF7 at the nexus of immune activation, cellular stress response, and tissue regeneration, highlighting its dual involvement in auditory and muscular aging. Its dysregulation may thus represent a common immunometabolic pathway through which chronic inflammation and impaired regenerative capacity drive the co-pathogenesis of ARHL and sarcopenia.

However, the transition from a research-based transcriptomic signature to an in vitro diagnostic test, such as a PCR-based blood assay, requires careful consideration. Key steps include the selection of an optimal biosource (e.g., peripheral blood mononuclear cells or serum cell-free RNA), assay optimization for sensitivity and specificity, and rigorous analytical validation per regulatory standards (e.g., FDA guidelines for in vitro diagnostics). A significant challenge will be demonstrating that the multi-gene RNA signature offers a clear advantage over or adds meaningful value to existing, simpler clinical parameters, thereby justifying the likely higher cost and complexity. Furthermore, the implementation of such a test would require defining clear clinical decision-making thresholds and integrating it into geriatric assessment workflows, potentially as a screening tool to identify high-risk individuals for early intervention.

Several limitations should be acknowledged. First, the reliance on murine transcriptomic data for HL modeling may limit translational relevance, given interspecies differences in auditory system biology. Second, the small sample size in certain cohorts (e.g., GSE6045 for HL, GSE38718 for ARS) increases the risk of overfitting and may limit the generalizability of the perfect AUC values observed. While we employed cross-validation and permutation tests to mitigate this concern, future validation in larger, prospective cohorts is essential. Third, it is important to emphasize that the biomarker identification in this study is based solely on transcriptomic data. While our multi-cohort, machine-learning approach robustly identifies hub genes at the mRNA level, these findings require further validation through protein-level assays (e.g., Western blot, ELISA, or immunohistochemistry) and functional studies to confirm their biological and clinical relevance. The absence of proteomic or functional validation is a limitation of the current study. Finally, the performance of these biomarkers may vary in more heterogeneous populations. Comorbidities such as chronic inflammatory conditions or liver disease could influence the expression of genes like SERPING1 or JUN. Polypharmacy, common in the elderly, is another potential confounder. Therefore, future studies must include covariate adjustment and subgroup analyses to ensure the biomarkers’ predictive power is independent and robust. Despite these challenges, the convergence of pathways related to inflammation and immune dysfunction in both conditions provides a strong biological rationale for these biomarkers. Their ultimate clinical utility may lie not as standalone diagnostics, but as part of a composite risk stratification tool that combines molecular markers with imaging and functional assessments to guide personalized management strategies for age-related decline.

Future work should focus on experimental validation of the proposed hub genes. Potential steps include: Quantitative real-time PCR to verify gene expression in independent human tissue samples (e.g., cochlear and muscle biopsies). Immunohistochemistry to assess protein expression and localization in relevant tissues. Prospective clinical studies to evaluate the utility of these biomarkers in serum or plasma samples using immunoassays. Functional studies using in vitro or in vivo models (e.g., gene knockdown or overexpression) to elucidate the mechanistic roles of key genes such as SERPING1 or IRF7 in HL and sarcopenia.

## 5. Conclusion

In conclusion, our study provides a transcriptomic-based ML framework for identifying shared diagnostic biomarkers for ARHL and sarcopenia. The proposed hub genes show strong diagnostic potential but await further experimental and clinical validation to confirm their utility as noninvasive biomarkers or therapeutic targets.

## Author contributions

**Conceptualization:** Ming Li.

**Data curation:** Ming Li.

**Formal analysis:** Ming Li.

**Funding acquisition:** Ming Li.

**Investigation:** Ming Li.

**Methodology:** Ming Li.

**Project administration:** Ming Li.

**Resources:** Ming Li.

**Software:** Ming Li.

**Supervision:** Ming Li.

**Validation:** Ming Li.

**Visualization:** Ming Li.

**Writing – original draft:** Ming Li.

**Writing – review & editing:** Ming Li.

## Supplementary Material


